# Electrical and Dielectrical Properties of Composites Based on Alumina and Cyclic Olefin Copolymers

**DOI:** 10.3390/ma17215349

**Published:** 2024-10-31

**Authors:** Eusebiu Ilarian Ionete, Artur Visse, Radu Dorin Andrei, Mirela Irina Petreanu, Stefan Ionut Spiridon, Roxana Elena Ionete

**Affiliations:** 1 National Research and Development Institute for Cryogenic and Isotopic Technologies-ICSI, 240050 Râmnicu Vâlcea, Romania; radu.andrei@icsi.ro (R.D.A.); irina.petreanu@icsi.ro (M.I.P.); ionut.spiridon@icsi.ro (S.I.S.); roxana.ionete@icsi.ro (R.E.I.); 2 Canoe Platform, CANOE-Le Centre Technologique Nouvelle Aquitaine Composites & Matériaux Avancés, Bât CHEMINNOV–ENSMAC, 33600 Pessac, France; visse@plateforme-canoe.com

**Keywords:** alumina, composites, conductivity, cryogenics, cyclic olefin copolymer, dielectrics

## Abstract

Understanding the performance of polymer dielectrics at different temperatures is becoming increasingly important due to the rapid development of electric cars, electromagnetic devices, and new energy production solutions. Cyclic olefin copolymers (COCs) are an attractive material due to their low water absorption, good electrical insulation, long-term stability of surface treatments, and resistance to a wide range of acids and solvents. This work focused on the dielectric and electrical properties of cyclic olefin copolymer (COC)/Al_2_O_3_ composites over a wide range of temperature and frequency domains, from room temperature to cryogenic temperatures (around 125 K). Permittivity, electrical conductivity, and electrical modulus are given consideration. A composite of up to 50% Al_2_O_3_ mixed with COC was prepared via a conventional melt-blending method. The final samples were formed in sheets and processed using injection and extrusion moldings. It was found that formulations with Al_2_O_3_ concentrations ranging from 10 to 50% resulted in higher electrical conductivity while maintaining the viscosity of the composite at a level acceptable for polymer-processing machinery. Our data show that COC/alumina composites present substantial potential as materials for high-frequency applications, even at the regime of cryogenic temperatures.

## 1. Introduction

Cyclic olefin copolymers (COCs) are a class of advanced amorphous thermoplastics distinguished by their unique combination of properties, which make them ideal for cutting-edge technological applications. These materials are synthesized via the copolymerization of cyclic monomers like norbornene with linear olefins such as ethene or propene, typically using metallocene-catalyzed polymerization techniques [1,2,3,4,5,6,7,8,9]. This allows for fine-tuning of the molecular structure and the resulting physical properties of the polymer [5,7].

COCs exhibit several key advantages, such as high glass transition temperatures (T_g_), typically ranging from 80 °C to 180 °C depending on their composition [6,10]. They also possess excellent optical transparency, low moisture absorption, and good resistance to a wide range of chemicals [6,11,12,13], making them ideal for applications in optical components, pharmaceutical packaging, and microfluidic devices [6,11,14,15,16,17,18]. Their high modulus of elasticity, typically between 1.5 and 2.5 GPa, combined with high impact resistance, renders them suitable for mechanically demanding applications [19,20]. Different methods for the preparation and characterization of structural and physical properties of COC nanocomposites, including various types of inorganic nanofillers such as silica [21], organo-clay [22], and polyhedral oligomeric silsesquioxanes (POSS) [23], have been studied. Dispersion of COC particles in various amounts in an epoxy matrix have also been reported [24]. So far, only a few works have been reported on the preparation and characterization of high-frequency dielectric COC composited with various types of conductive fillers [17,25,26,27].

However, the intrinsic dielectric properties of COCs may limit their use in certain high-frequency and high-temperature environments. As the demand for materials with superior dielectric performance grows, particularly in fields such as electronics, energy storage, and telecommunications, researchers have focused on enhancing these properties through the incorporation of inorganic fillers such as alumina (Al_2_O_3_). Alumina is well-known for its high dielectric constant (8.5–10.5), thermal stability, and excellent electrical insulation [28,29,30]. These properties make it an attractive filler material for improving both the dielectric and mechanical properties of COCs. The incorporation of alumina into COC matrices has been shown to significantly enhance their dielectric properties. For example, composites with alumina concentrations of 10–50 wt% have exhibited increases in dielectric constant by up to 30% over neat COCs, with minimal dielectric losses even at frequencies as high as 1 MHz [31]. This enhancement is particularly relevant for applications such as capacitors and high-frequency devices, where stable dielectric performance across a wide temperature and frequency range is critical [28].

In addition to dielectric improvements, the mechanical properties of COC/alumina composites are also enhanced. Studies on polymer composites reinforced with alumina, such as epoxy and unsaturated polyester matrices, show increases in mechanical properties; for example, adding alumina can enhance tensile strength and impact resistance, as well as flexural properties, in some systems [28,32]. The high thermal stability of alumina also contributes to improved thermal resistance in COC composites, enabling them to maintain mechanical integrity and dielectric performance at temperatures as high as 250 °C [33]. This makes them suitable for high-temperature applications in electronics and energy systems.

Previous research on other COC composites has provided valuable insights into how filler materials affect the properties of the polymer. For instance, COC/graphite composites have demonstrated increased electrical conductivity [34,35], while COC/silica composites have shown enhancing properties like stiffness, tensile strength, and thermal stability [36,37]. However, fewer studies have explored the specific advantages of alumina as a filler in COC matrices. Alumina’s potential to enhance dielectric properties, especially in high-frequency and broad temperature applications, has been observed, though its use in cryogenic conditions remains relatively underexplored. Research indicates that alumina ceramics perform well in maintaining low dielectric losses and stable dielectric constants across various frequencies and elevated temperatures, such as up to 550 °C. However, there are limited data specifically addressing the behavior of alumina-enhanced composites under cryogenic conditions, which could offer new avenues for material performance in extreme environments [33,38,39]. These studies highlight that alumina’s dielectric properties are sensitive to factors such as microstructure, purity, and the method of preparation, influencing its ability to perform under a wide range of temperatures and frequencies. This supports the potential for future research to explore alumina’s role in cryogenic applications.

This study aims to address this gap by systematically investigating the dielectric, electrical, and mechanical properties of COC/alumina composites across a broad range of alumina loadings (10% to 50%) and temperatures, including cryogenic temperatures down to 125 K. The composites are synthesized using melt-blending techniques, and their properties are evaluated to identify the optimal balance between dielectric performance, mechanical integrity, and processability. By exploring alumina concentrations beyond those previously studied, this research seeks to contribute to the development of COC-based composites for use in high-frequency, high-temperature, and cryogenic applications.

## 2. Materials and Methods

### 2.1. Materials

Cycloolefins copolymer (COC, TOPAS^®^ 6017s-04) resins were purchased from TOPAS Advanced Polymers Inc. (Raunheim, Germany). Spherical alumina, Al_2_O_3_, was purchased from DENKA (Tokyo, Japan). The product, Denka Spherical Alumina (trade name DAM-45), is a high-sphericity alumina, developed by Denka, using a series of unique high-temperature melting technologies.

### 2.2. Preparation of COC Film

#### 2.2.1. Compounding

COC pellets and alumina powder were dried in an oven at 100 °C for 4 h under air atmosphere. The different compounds (Table 1) are produced by using a twin-screw conical micro compounder (XPLORE MC 15 HT, Xplore Instruments B.V., Sittard, The Netherlands).

For each mixing batch, 15 g of material (a combination of COC and alumina) was weighed and progressively introduced into the micro-compounder hopper. The materials were mixed for 3 min, after which the micro-compounder by-pass was opened. The blend was then extruded through the micro-compounder die and cooled on a conveyor belt. Once cooled, the blends were cut into pellets.

#### 2.2.2. Injection

The COC/alumina pellets were dried in an oven at 100 °C for 4 h. The dried blends were then injection-molded to produce sheets measuring 25 × 25 × 0.8 mm.

### 2.3. Analytical Investigation

Broadband dielectric measurements were conducted using a Novocontrol Concept 40 high-resolution Alpha dielectric analyzer, Novocontrol Technologies GmbH & Co. Montabaur, Germany covering a frequency range from 10^−1^ Hz to 10^7^ Hz and a temperature range from 150 °C to 80 °C. The process was supported by a Quattro Cryosystem temperature controller, Novocontrol Technologies GmbH & Co. ensuring temperature stability within 0.3 K, in a dry nitrogen atmosphere. This analyzer is specifically designed for materials research, enabling measurements across a wide frequency spectrum.

**Figure 1:**
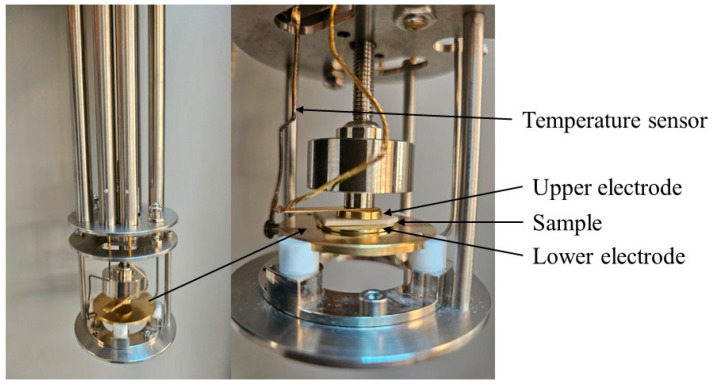
Image of the capacitor cell within the spectrometer.

The polyelectrolyte thin film was positioned between two polished gold electrodes, measuring 25 mm and 15 mm, forming a parallel plate capacitor cell. These sandwich samples (Figure 1) were then placed in the spectrometer (Novocontrol Technologies GmbH & Co. KG, Montabaur, Germany). Dielectric measurements were performed isothermally in 10 K increments during both cooling and heating phases to ensure reproducibility, using an AC voltage amplitude of 0.1 V across a broad temperature range (from 123 to 353 K).

Thermal analysis was performed using a Simultaneous Thermal Analyzer—STA 449 F3 Jupiter (Neztsch-Gerätebau GmbH, Selb, Germany). The samples were subjected to a thermal program involving a temperature increase at a rate of 10 °C/min, from ambient temperature up to 800 °C. Each sample, weighing approximately 20–30 mg, was placed in an alumina (Al_2_O_3_) crucible and maintained under a nitrogen inert atmosphere (100 mL/min) throughout the entire thermal program. During thermal degradation, the sample mass (mass%), heat flow (mV/mg), and temperature (°C) were recorded.

X-ray Diffraction (XRD) measurements were carried out using a Rigaku MiniFlex600 X-ray diffractometer (Rigaku, Tokyo, Japan), equipped with a Cu K-α X-ray source (λ = 1.541838 Å). Scans were recorded over a 2θ range of 15° to 80° at high resolution, with a scanning speed of 2.0°/min and a step size of 0.05°. Signal processing and analysis were performed using PDXL2 software (Rigaku, Tokyo, Japan), supplemented by Crystallography Open Database (COD) cards referenced from the relevant literature card numbers 9009674 for Al_2_O_3_ [40] and 2107302 for Al_2.4_O_3.6_ [41].

### 2.4. Determination of Dielectric Features

#### 2.4.1. Dielectric Constant

The dielectric response of the COC/alumina composites, named complex dielectric permittivity, $\varepsilon^{*}\left(\omega \right)$, was measured as a function of frequency and temperature, focusing on both the real and imaginary components, and expressed as follows:

(1) $$\varepsilon^{*}\left(\omega \right)=\varepsilon^{\prime }\left(\omega \right)-j\varepsilon^{\prime \prime }\left(\omega \right),$$

where $\varepsilon^{\prime }\left(\omega \right)$ is the relative permittivity (real part of the dielectric constant, which measures the material’s ability to store electrical energy as polarization in an electric field), $\varepsilon^{\prime \prime }\left(\omega \right)$ is the dielectric loss (imaginary part of the dielectric constant, representing energy losses due to dielectric relaxation and conductivity), and ω is the angular frequency of the applied electric field [42,43]. These two components define how effective the composite will be in applications such as high-frequency electronics.

The real part of the permittivity, $\varepsilon^{\prime }\left(\omega \right)$, was determined using capacitance measurements under varying frequency conditions. The measurements were conducted in the frequency range of 10^−1^ to 10^7^ Hz, with temperature-controlled experiments to assess the temperature dependence of the dielectric response. The dielectric constant, $\varepsilon^{\prime }\left(\omega \right)$, was calculated using this Equation:

(2) $$\varepsilon^{\prime }\left(\omega \right)=\frac{C\left(\omega \right)\cdot d}{\varepsilon_{0}\cdot A},$$

where $C\left(\omega \right)$ is the measured capacitance, d is the sample thickness, A is the surface area of the sample, and $\varepsilon_{0}$ is the permittivity of free space (≈8.854 × 10^−12^  F/m).

The dielectric loss, represented by $\varepsilon^{\prime \prime }\left(\omega \right)$, was evaluated to understand energy dissipation mechanisms in the composite. This loss is primarily attributed to the dielectric relaxation processes and the interaction between the COC matrix and the dispersed alumina particles. The imaginary component was calculated using the dissipation factor, $\tan\delta \left(\omega \right)$, as follows:

(3) $$\epsilon^{\prime \prime }\left(\omega \right)=\epsilon^{\prime }\left(\omega \right)\cdot \tan\delta \left(\omega \right),$$

#### 2.4.2. Electric Modulus

The electrical modulus is an essential tool in understanding the dielectric relaxation and conduction mechanisms in ion-conducting materials, as it has the advantage of suppressing electrode polarization effects. In this study, the electrical modulus, $M^{*}(\omega )$, of the cyclic olefin copolymer (COC)/alumina (Al_2_O_3_) composites was experimentally investigated across a range of frequencies and temperatures to elucidate the relaxation dynamics of the system.

The complex electrical modulus is defined as the reciprocal of the complex dielectric permittivity [44]:

(4) $$M^{*}\left(\omega \right)=\frac{1}{\varepsilon^{*}\left(\omega \right)}=M\prime \left(\omega \right)+jM\prime \prime \left(\omega \right),$$

where $M^{\prime }(\omega )$ is the real part of the electrical modulus, representing the energy storage or elastic response of the material, and $M^{\prime \prime }(\omega )$ is the imaginary part, associated with energy dissipation due to dielectric relaxation.

The frequency-dependent electrical modulus of COC/alumina composites was obtained using the measured dielectric permittivity. The real and imaginary components of the electrical modulus were analyzed to provide insight into the relaxation times and the distribution of relaxation processes in the material.

To extend the scientific data concerning the frequency dependence of the electrical modulus components $M\prime \left(\omega \right)$ and $M\prime \prime \left(\omega \right)$, it is important to understand the relationship between these modulus values and the impedance of the system. The electrical modulus formalism is particularly useful for characterizing dielectric relaxation phenomena in composite materials, like COC/alumina systems, where the interaction between the matrix and fillers can significantly alter the electrical properties.

The expressions for the real and imaginary parts of the electrical modulus are calculated using the impedance components Z′ and Z″ and the angular frequency ω as follows:

(5) $$M\prime \left(\omega \right)=\omega C_{0}Z\prime \prime \left(\omega \right)\;\mathrm{and}\;M\prime \prime \left(\omega \right)=\omega C_{0}Z^{\prime }\left(\omega \right),$$

where $Z^{\prime }\left(\omega \right)$ is the real part of the complex impedance, $Z^{\prime \prime }\left(\omega \right)$ is the imaginary part of the complex impedance, and $C_{0}$ is the vacuum capacitance, given by $C_{0}=\varepsilon_{0}\cdot A/d$, with $\varepsilon_{0}$ as the permittivity of free space, *A* the area of the electrode, and *d* the thickness of the sample.

The use of the electrical modulus formalism allows for a deeper understanding of the dielectric properties of COC/alumina composites, especially in identifying the relaxation dynamics. By analyzing $M\prime \left(\omega \right)$ and $M\prime \prime \left(\omega \right)$, one can assess how the material stores and dissipates electrical energy across a range of frequencies.

## 3. Results

### 3.1. Dielectric Constant Analysis

The dielectric properties of COC6-based samples were studied using broadband dielectric measurements in the frequency range of 10^−1^ to 10^7^ Hz and over a temperature range from 135 to 353 K. Permittivity of the samples gradually decreases with increasing frequency—see Figure S1 (Supplementary Material). This occurs because, at high frequencies, the dipoles’ rotation and movement cannot keep pace with the changes in the electric field, with some dipoles even ceasing to reorient. Consequently, the addition of Al increases the permittivity of the COC6 samples, resulting in higher permittivity compared to pure COC6. As the Al content in COC6-Al samples increases, their permittivity also increases [45]. However, all types converge slightly with increased frequency, indicating that the additive effect is more significant at lower frequencies.

The rate at which the dielectric constant decreases appear to vary among the different COC6 types, and this is more visible at 353 K: (a) COC6-50 shows a steady decrease, indicating a slightly higher sensitivity to frequency changes compared to other types; (b) COC6-10 to COC6-40: The decrease is also apparent but not as pronounced. These variants maintain a relatively stable value compared to COC6-50; (c) Base COC6: the dielectric constant is almost flat across all frequencies, suggesting that the unmodified material is least affected by frequency changes.

There is a trade-off between achieving a higher dielectric constant and maintaining stability across different frequencies. The highest additive content (COC6-50) yields a higher dielectric constant but comes with reduced stability at higher frequencies.

Also, the dielectric constant tends to increase with increasing temperature. For instance, at 353 K, the dielectric constant values are generally higher compared to those at 123 K. This indicates that the dipoles in the material have higher energy at elevated temperatures, which increases their ability to align with an electric field, resulting in higher polarization.

Comparing trends for the specific COC6 variants, it can be observed that the dielectric constant for COC6 is consistently lower across all temperatures compared to the modified variants (e.g., COC6-50). The trend is flatter for lower temperatures (e.g., 123 K), which implies a higher stability, while at higher temperatures like 353 K, the dielectric constant starts at a higher value and drops more with frequency. At 353 K, the dielectric constant starts above 3.9 and decreases more rapidly as frequency increases, whereas at lower temperatures like 123 K, the starting value is around 3.7, with a more gradual decline.

The sensitivity to temperature is more pronounced in modified COC6 variants (e.g., COC6-50, COC6-40). They show a higher dielectric constant at higher temperatures (353 K and 303 K) and a steeper decline with increasing frequency. At these temperatures, the rate of decline in dielectric constant with frequency is sharper, indicating that the material’s polarization decreases more quickly. This could be due to increased thermal agitation at higher temperatures, which makes it harder for dipoles to keep up with the electric field at higher frequencies. The dielectric constants at lower temperatures (173 K and 123 K) are generally lower, but the rate of change with frequency is more stable. This indicates that the polarization mechanism is less affected by frequency variations. In contrast, the base COC6 is less sensitive, showing smaller differences across temperatures.

**Figure 2:**
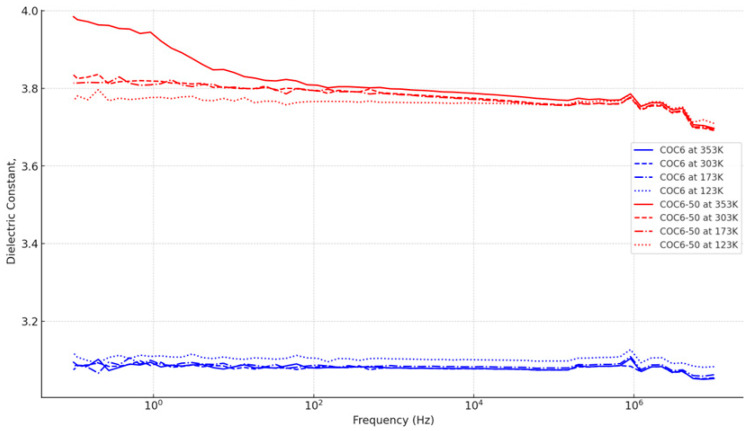
Dielectric constant against frequency for the base COC6 and the most modified variant (COC6-50) across temperatures (353 K, 303 K, 173 K, and 123 K).

Figure 2 summarizes these trends visually. COC6-50 has a clear dependence on temperature, with higher dielectric constants at higher temperatures and a sharper decrease with frequency. This suggests that increased temperatures enhance the dipole alignment but also make the material more susceptible to losing alignment at high frequencies. On the other hand, COC6 is more stable across temperatures, making it a better choice where temperature stability is critical.

Also, the dielectric constant values for all COC6 variants are relatively stable across the frequency range at 123 K. There is minimal change in the dielectric constant, which suggests that the polarization mechanism is less affected by frequency changes at this lower temperature. The base COC6 material has the lowest dielectric constant, starting around 3.1 and remaining stable. This indicates minimal enhancement without additives. As the number after COC6 increases (e.g., COC6-10, COC6-20), the dielectric constant also increases. This shows the impact of incremental modifications, which enhance the material’s ability to store electrical energy.

The stability of the dielectric constant across different frequencies at 123 K indicates that the dipoles in these materials are not significantly affected by the oscillating electric field at this low temperature, with COC6-50 offering the highest dielectric response. This makes COC6 materials suitable for applications where temperature is low, and a stable dielectric response is required.

The dielectric loss of the COC films as a function of temperature suggests excellent dielectric stability (Figure S2—Supplementary Material). As the temperature increases, the density of non-polar and weakly polar polymers gradually decreases, with this effect becoming more pronounced near the glass transition temperature (T_g_), leading to a decrease in the dielectric constant. However, due to the increased motion of molecular chains, the value of $\tan\delta \left(\omega \right)$ increases. Notably, the $\tan\delta \left(\omega \right)$ of the COC film remains nearly constant across the entire temperature range, indicating that the film can function up to 353.15 K. The addition of Al makes the COC6 crystal more compact, minimizing flaws in the film and enhancing the distortion of the local electric field. The dielectric loss tangent increases with increasing frequency because the polarization of the samples cannot keep pace with the changes in the external electric field [46].

Dielectric losses typically refer to the heat generated in dielectric materials when subjected to an alternating electric field, due to the internal consumption of electrical energy. A lower dissipation factor indicates better performance of a dielectric material in charge storage applications [46]. For conductive polymer composites, dielectric loss primarily results from two effects: direct current (DC) conductance and interfacial polarization. At low frequencies, DC conductance loss predominates, whereas at high frequencies, interfacial polarization loss becomes more significant [47]. In the materials we investigated, the dielectric dissipation factor shows a slight increase with increasing Al_2_O_3_ content.

**Figure 3:**
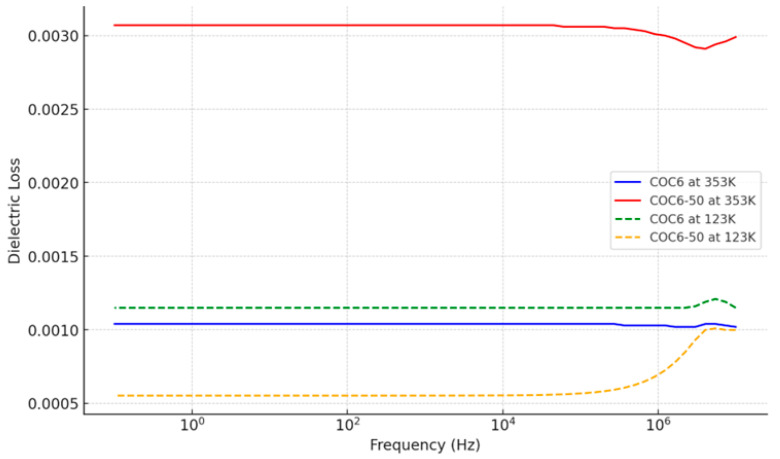
Dielectric loss for COC6 and COC6-50 at the lowest and highest investigation temperatures.

As observed from Figure 3, at higher temperatures (353.15 K), the dielectric loss is generally higher for both COC6 and COC6-50, while at lower temperatures (123.15 K), the dielectric loss decreases, and the difference between COC6 and COC6-50 becomes more pronounced. Across the frequency spectrum, the dielectric loss shows relatively consistent behavior with small variations at specific frequencies.

The increase in dielectric loss due to adding Al_2_O_3_, from COC6 to COC6-50, is more pronounced at higher temperatures (e.g., 353 K). At higher temperatures, the molecular mobility and polarization processes are more active, which leads to increased energy loss when more alumina powder is added. At lower temperatures (e.g., 123 K), the dielectric loss for both COC6 and COC6-50 is reduced.

Overall, adding Al_2_O_3_ to COC6 has a more significant impact on increasing dielectric loss at higher temperatures (e.g., COC6-50 shows a higher dielectric loss compared to COC6 at 353 K), which suggests that the added components introduce mechanisms for increased energy dissipation.

### 3.2. Electrical Modulus Analysis

Complex impedance spectroscopy is employed to characterize the electrical properties of materials, highlighting the dynamic behavior of charge carriers in interfacial regions within ionic, insulating, semiconducting, or mixed ionic–electronic materials [48]. The acquired response data are represented using parameters such as complex permittivity and dielectric modulus. The electrical modulus is particularly useful for determining the relaxation dynamics in ion-conducting materials, as it has the advantage of suppressing electrode polarization effects.

**Figure 4:**
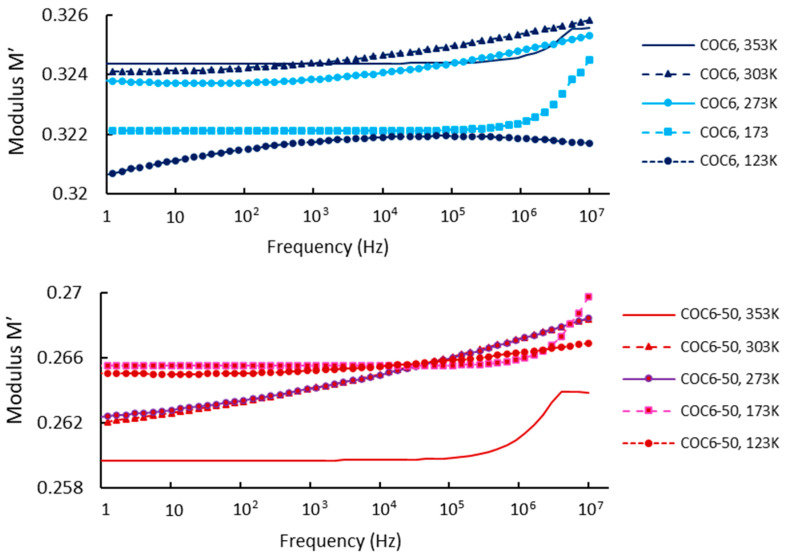
Modulus *M′*: comparison between COC6 and COC6-50 at different temperatures.

Regarding our results, Figure 4 shows the real part of the modulus, *M′*, as a function of frequency at different temperatures, covering data for base sample (COC6) and the sample with 50% Al_2_O_3_ addition (COC6-50). Based on these values, it can be observed that at low frequencies and low temperatures, *M′* remains constant, indicating that electrode polarization has a negligible and consistent effect on *M′* [49]. However, at higher frequencies, *M′* begins to increase slightly, which may be attributed to a conductivity-relaxation process occurring at those frequencies.

At higher temperatures (e.g., 353 K), the modulus (*M′*) is lower for the COC6 (base sample), indicating a more relaxed or less rigid structure, while with the decrease in temperature, the modulus increases, indicating increased stiffness or reduced segmental motion of the material. For the blends (e.g., COC6-50), the *M′* is generally lower at higher temperatures compared to the base sample (COC6), which suggests that the presence of added Al_2_O_3_ decreases the overall modulus at elevated temperatures. As for COC6, the *M′* increases as the temperature decreases, but the values remain higher than those of COC6, especially at lower temperatures (e.g., 123 K). This indicates that the added Al_2_O_3_ to COC, more evident in the COC6-50 sample, enhances rigidity at low temperatures, likely due to the interactions between the additives and the polymer matrix.

In general, the presence of additives (Al_2_O_3_) to COC samples affects its temperature-dependent modulus by reducing the modulus at high temperatures, which may provide better flexibility, while increasing stiffness at lower temperatures, suggesting improved structural stability in colder conditions.

**Figure 5:**
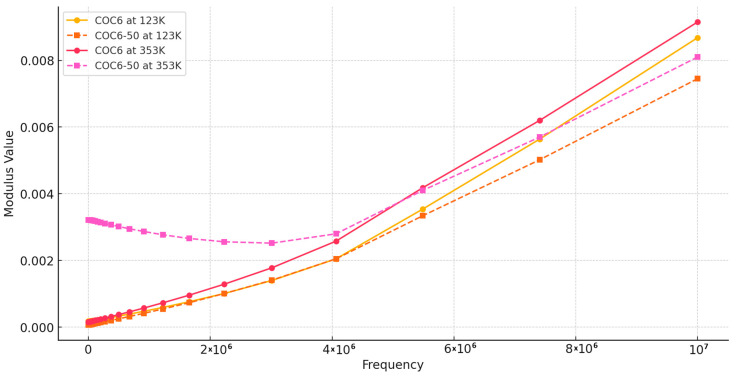
Modulus *M”*: comparison between COC6 and COC6-50 at the lowest and highest testing temperatures (123 K and 353 K, respectively).

Figure 5 illustrates the frequency dependence of *M″*, the imaginary part of the modulus, representing the energy dissipation within the materials when subjected to an alternating stress or electric field. The observed trends in *M″* across different temperatures help characterize the viscoelastic behavior and energy dissipation mechanisms, which are essential for optimizing materials for cryogenic applications. For all the materials samples (from COC6 to COC6-50) at both lower (e.g., 123 K) and higher (e.g., 353 K) temperatures, *M″* generally increases with increasing frequency (Figure S4—Supplementary Materials). This trend indicates that at higher frequencies, the energy dissipation within the material is greater; it is typical for viscoelastic materials, where higher frequencies correspond to greater internal friction and less ability for molecular chains to relax in response to the rapidly changing external field.

Comparing COC6 versus COC6-50, was observed that COC6 highlight slightly higher *M″* values compared to COC6-50 across most temperatures and frequencies. This suggests that COC6 has greater energy dissipation than COC6-50, possibly due to differences in molecular structure or cross-linking density, which affects the material’s ability to dissipate energy through molecular motion.

At low frequencies, *M″* tends to be smaller. This is because, at low frequencies, the applied external field or stress allows more time for the material’s dipoles or molecular segments to align with the field.

In a cryogenic temperature context, molecular mobility is significantly restricted due to reduced thermal energy. The relaxation processes are slower, and many molecular motions are “frozen”. Thus, at these temperatures, the material shows relatively low losses at low frequencies.

### 3.3. Thermal Analysis of COC Compounds

**Figure 6:**
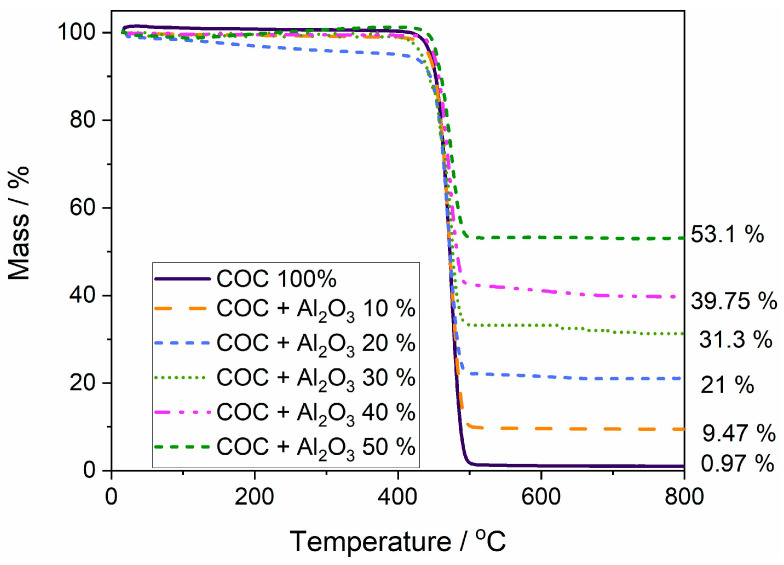
Overlapped thermal gravimetric curves for polymer sample (COC 100%) and composite (COC + x% Al_2_O_3_) samples.

The mass loss curves (Figure 6) were analyzed for polymer samples without alumina (COC6) and with 10% (COC6-10), 20% (COC6-20), 30% (COC6-30), 40% (COC6-40), and 50% (COC6-50) alumina (Al_2_O_3_) added. COC6 shows thermal stability up to approximately 415 °C; beyond this point, a rapid decrease in mass is observed, indicating decomposition. The mixed samples (COC6-10 to COC6-50) display improved thermal stability compared to pure COC (COC6). The thermal decomposition starts at higher temperatures, reaching up to around 435 °C for the COC sample with 50% Al_2_O_3_. The remaining mass after heating varies significantly: the COC + Al_2_O_3_ 50% sample has the highest residual mass at about 53.1%, whereas COC + Al_2_O_3_ 10% retains about 9.47%.

The incorporation of Al_2_O_3_ in increasing percentages effectively enhances the thermal stability of the COC samples. The higher content of Al_2_O_3_ results in greater residue (Figure S3—Supplementary Material), indicating a more thermally stable composite material compared to the pure polymer. The pure COC leaves almost no residue, whereas COC + Al_2_O_3_ 50% leaves more than half of the mass (53.1%). This suggests that Al_2_O_3_ is not only thermally stable but also plays a role in protecting the polymer matrix during decomposition. The addition of Al_2_O_3_ acts as a reinforcing filler, improving the overall thermal resistance of the polymer blends. This is likely due to the ceramic-like nature of Al_2_O_3_, which has high thermal stability and can act as a heat sink, thus delaying the breakdown of the polymer matrix.

**Figure 7:**
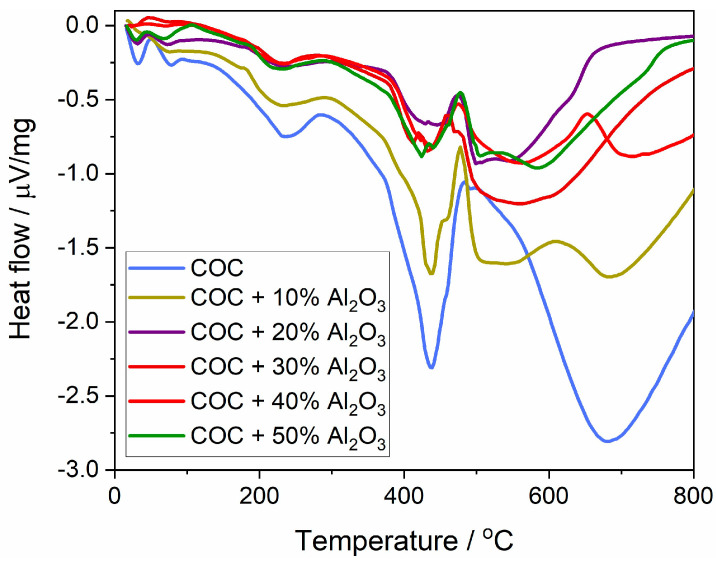
Heat flux variation curves for cyclic olefin copolymer (COC) samples with varying proportions of alumina (0%, 10%, 20%, 30%, 40%, and 50% Al_2_O_3_).

The variation in thermal flow (Figure 7) represents the heat absorbed or released during phase transformation processes at different temperatures throughout the thermal analysis. The first transition occurs with heat absorption at around 230 °C, attributed to the softening/melting of the crystalline phase of the semi-crystalline polymer. This melting point appears relatively consistent across all samples regardless of Al_2_O_3_ content, indicating that the incorporation of Al_2_O_3_ does not significantly affect the melting temperature of the crystalline phase.

The next significant thermal transition involves polymer chain degradation, resulting in a sharp absorption curve. For the COC and COC + 10% Al_2_O_3_ samples, a sharp peak is observed at 436 °C, indicating that the polymer chains undergo a rapid degradation at this temperature, which results in significant heat absorption. For the 40% and 50% Al_2_O_3_ composite samples, a broader absorption peak occurs between 380 and 480 °C, suggesting a more gradual thermal degradation, which can be attributed to the protective effect of higher Al_2_O_3_ content.

The heat flow values for the COC + Al_2_O_3_ composites generally show less dramatic fluctuations compared to the pure COC. This means that adding Al_2_O_3_ reduces the extent of thermal energy changes during phase transitions, enhancing the overall thermal performance of the composite material.

**Figure 8:**
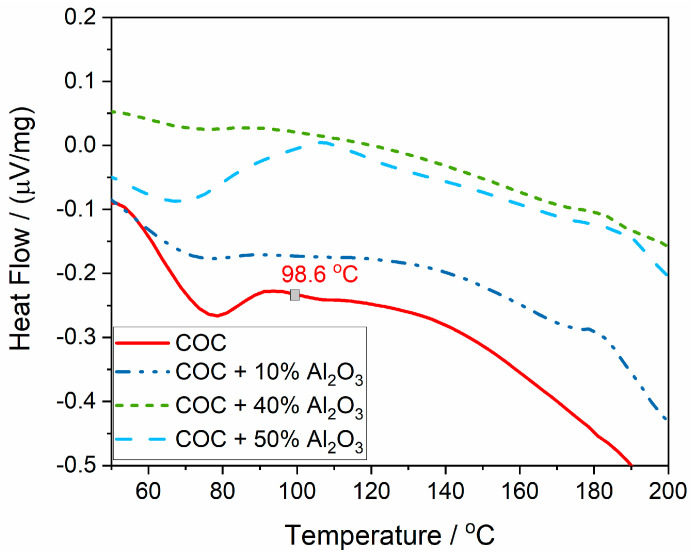
The glass transition point (T_g_), determined from the heat flow variation curve.

The glass transition temperature (T_g_), representing the temperature at which the polymer transitions from a glassy (rigid) state to a more rubbery, flexible state, is evident for the polymer without alumina, but it could not be accurately determined for the composite samples (Figure 8). For the COC pure sample (COC6), T_g_ was determined as the inflection point in the heat flux variation curve and is found to be 98.6 °C.

The presence of alumina seems to blur the T_g_ signal, suggesting that the alumina filler interferes with the polymer’s glass transition process. The polymer chains in the composite are likely restricted by the Al_2_O_3_ particles, leading to a less distinct change in heat flow that typically marks the T_g_. The reduction in clarity of the T_g_ with increasing alumina content indicates stronger interactions between the polymer and the filler, which could be beneficial for improving the thermal stability of the material. However, it also makes it more challenging to determine the exact thermal transitions of the composite.

**Figure 9:**
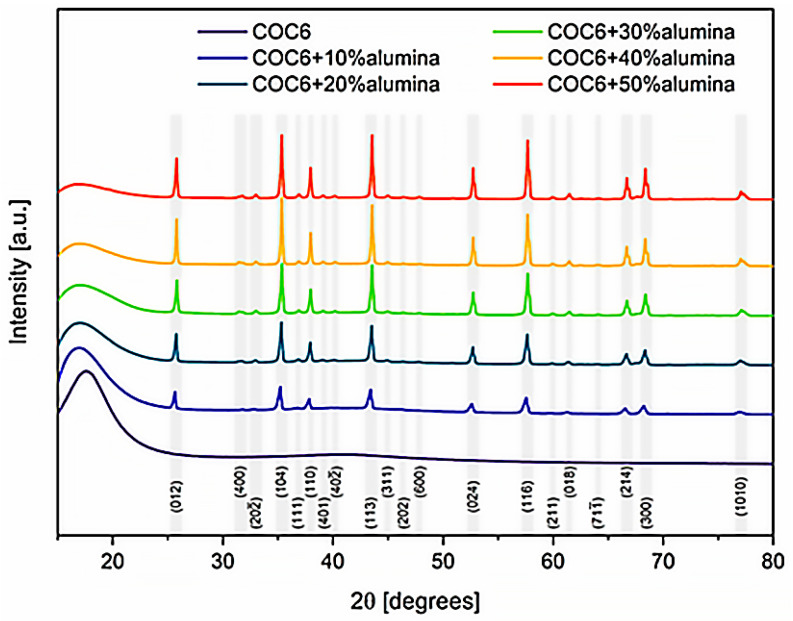
Comparative XRD spectra for all COC6 (cyclic olefin copolymer) composites with added alumina particles vs. pristine COC6. Miller indices for major peaks correspond to the prevailing alumina phase as identified during analysis (Al_2_O_3_ vs. Al_2.4_O_3.6_). The initial broad peak around 16° corresponds to the amorphous COC6 phase.

To provide insights into the crystalline structure of the cyclic olefin copolymer (COC6) and its composites with different percentages of alumina (Al_2_O_3_) particles, an XRD investigation has been performed. The X-ray diffraction (XRD) spectra (Figure 9) revealed the coexistence of two distinct alumina phases with varying proportions in each analyzed composite sample: Al_2_O_3_ (hexagonal R-3c), identified using COD card number 9009674 [40] and Al_2.4_O_3.6_ (C12/mL), based on COD card number 2107302 [41].

Angular correction of lattice constants after Rietveld refinement provided the adjusted lattice parameters as follows: for the Al_2_O_3_ phase, *a* = *b* = 4.74 ± 0.01 Å, *c* = 12.96 ± 0.01 Å, *V* = 253 ± 1 Å^3^, *α* = β = 90.0°, *γ* = 120.0°; and for the Al_2.4_O_3.6_ phase, *a* = 11.48 ± 0.1 Å, *b* = 2.96 ± 0.1 Å, *c* = 5.24 ± 0.1 Å, *V* = 180 ± 5 Å^3^, α = *γ* = 90.0°, β = 104 ± 1°. Variations in lattice parameters are attributed to the differing percentages of alumina phase content. However, the calculated strain using the Williamson–Hall method remained low, not exceeding 1.3% for the Al_2.4_O_3.6_ phase in the case of 10% alumina addition and staying below 0.25% in all other cases.

This XRD analysis confirms that the incorporation of alumina, especially in higher concentrations (30%, 40%, and 50%), clearly changes the overall diffraction pattern; it has a significant effect on the material’s structural properties, converting the overall phase from predominantly amorphous (for COC6) to partially crystalline, depending on the amount of alumina added.

## 4. Conclusions

This study aimed to investigate the dielectric and thermal performance of cyclic olefin copolymer (COC6) films, both in their pure form and combined with varying concentrations of alumina (Al_2_O_3_) ranging from 10% to 50%, to assess their suitability for cryogenic applications. The films, with a thickness of 0.8 mm, were successfully fabricated using the injection-casting method and were characterized for their dielectric and thermal properties.

The experimental results demonstrated that the incorporation of alumina significantly enhances the dielectric and thermal performance of the COC6 matrix. The addition of Al_2_O_3_ has a pronounced effect on the dielectric constant, which increases as the alumina concentration rises, particularly at elevated temperatures. At higher frequencies, a modest decrease in the dielectric constant was observed, indicating the frequency-dependent nature of dipole alignment in the material. The dielectric loss exhibited a dual behavior, decreasing at low temperatures, which is beneficial for cryogenic applications, but increasing at higher temperatures due to enhanced polarization effects. This temperature sensitivity underscores the influence of Al_2_O_3_ as a filler, with the dielectric loss also demonstrating a decrease with increasing frequency and an increase as the alumina content rose.

Incorporating alumina also significantly improved the thermal stability of the COC6 composites. As the alumina content increased, the thermal decomposition temperature of the composites rose, confirming the protective effect of the filler on the polymer matrix. The structural analysis, performed through X-ray diffraction, further highlighted a transition from an amorphous to a partially crystalline phase with increasing alumina content, which contributed to the enhanced dielectric and thermal properties of the material.

The results of this study suggest that the type and concentration of filler material play a crucial role in determining the dielectric, thermal, and mechanical properties of COC6-based composites. The addition of Al_2_O_3_ not only improved dielectric constant and reduced dielectric loss at low temperatures but also enhanced the overall thermal performance of the composites. These findings establish a foundation for future studies aimed at optimizing filler materials for high-frequency and cryogenic applications. Further work should include mechanical testing and additional optimization of filler compositions to refine the balance between dielectric performance and processability, ensuring the development of advanced materials for high-performance applications.

## Figures and Tables

**Table 1:** Blends compositions, compounding, and injection parameters.

Sample Code	Composition	Injection Temperature (°C)	Barrel Temperature (°C)	Injection Pressure (bar)	Mold Temperature (°C)	Screw Speed (rpm)	Mixing Time (min)
COC6	COC	270	290	500	140	150	3
COC6-10	COC 90%w + Al_2_O_3_ 10%w	275	290	500	140	150	3
COC6-20	COC 90%w + Al_2_O_3_ 20%w	275	290	500	140	150	3
COC6-30	COC 90%w + Al_2_O_3_ 30%w	275	290	500	140	150	3
COC6-40	COC 90%w + Al_2_O_3_ 40%w	275	290	500	140	150	3
COC6-50	COC 90%w + Al_2_O_3_ 50%w	275	290	500	140	150	3

## Data Availability

The raw data supporting the conclusions of this article will be made available by the authors upon request.

## Supplementary Materials

The following supporting information can be downloaded at: https://www.mdpi.com/article/10.3390/ma17215349/s1, Figure S1. Dielectric constant of COC6-based samples as a function of frequency, at 123 K, 173 K, 273 K, 303 K, and 353 K, Figure S2. Dielectric loss of COC6-based samples as a function of frequency, Figure S3. Final residue percentage of alumina, Figure S4. Trends of *M′*, in terms of frequency and temperature.
