# Supplementary material for: Electrical and Dielectrical Properties of Composites Based on Alumina and Cyclic Olefin Copolymers
Source: Materials (Basel). 2024 Oct 31;17(21):5349. doi: 10.3390/ma17215349 (PMC11547529; doi:10.3390/ma17215349)
Supplement: Supplementary file 1 [file materials-17-05349-s001.zip › materials-3267061-supplementary.pdf]

Supplementary material

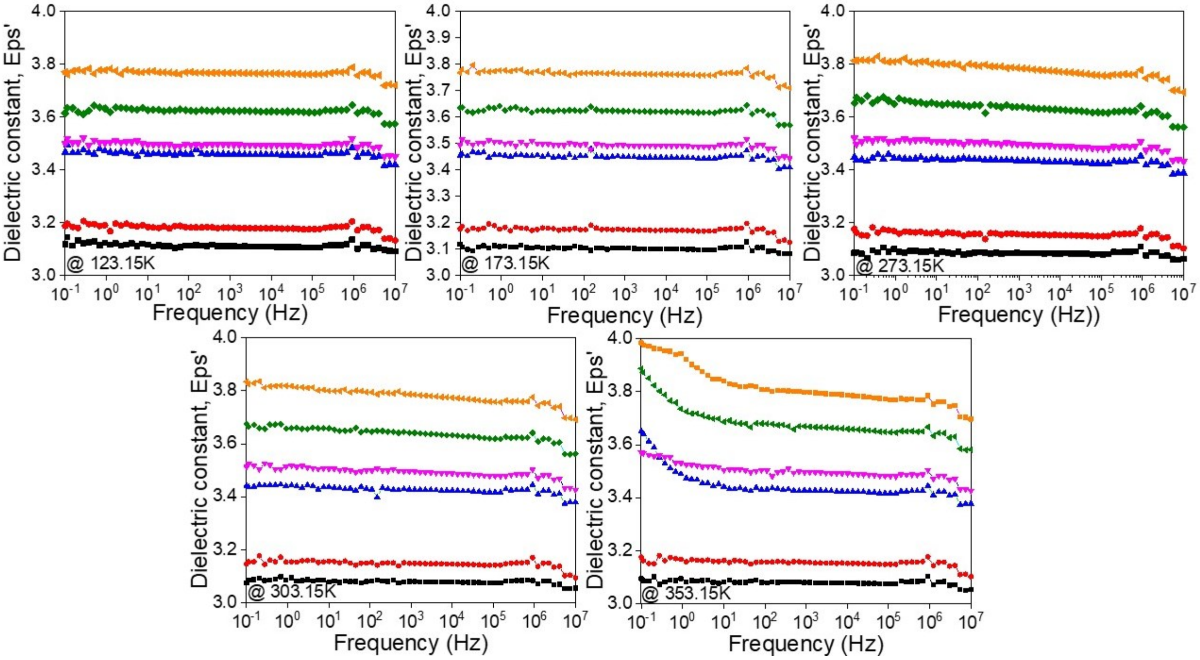


**Figure S1.** Dielectric constant of COC6-based samples as a function of at 123 K, 173 K, 273 K, 303 K and 353 K frequency (■ – COC6; ● – COC6-10%Al; ▲– COC6-20%Al; ▼– COC6-30%Al; ♦– COC6-40%Al; ◄– COC6-50%Al).

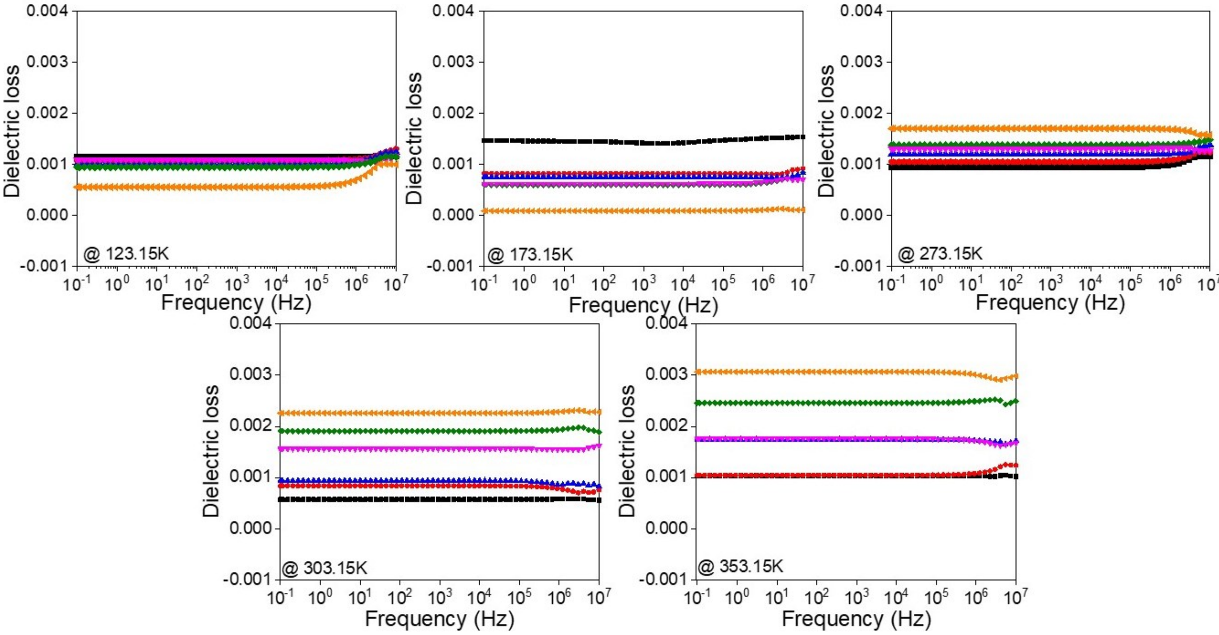


**Figure S2.** Dielectric loss of COC6-based samples as a function of frequency (■ – COC6; ● – COC6-10%Al; ▲– COC6-20%Al; ▼– COC6-30%Al; ♦– COC6-40%Al; ◄– COC6-50%Al)

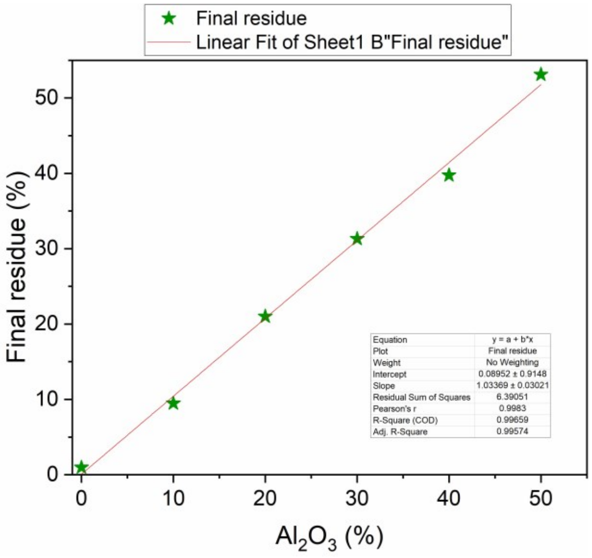


**Figure S3.** Final residue percentage of alumina

Figure 3S shows the distribution of the final residue as a function of the percentage of alumina in the COC matrix - the variation of final residue values depending on % of alumina fits well a linear curve (0.99659 R-square). The percentage of the final residue corresponds to the percentage of alumina, with a linear variation, as alumina does not thermally degrade at the temperatures used in the investigation.

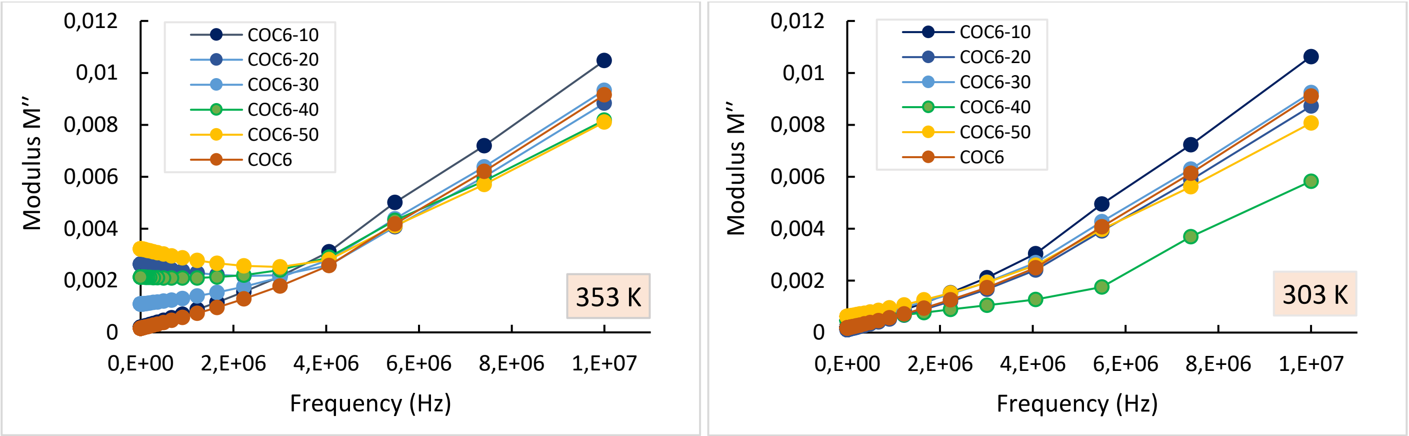

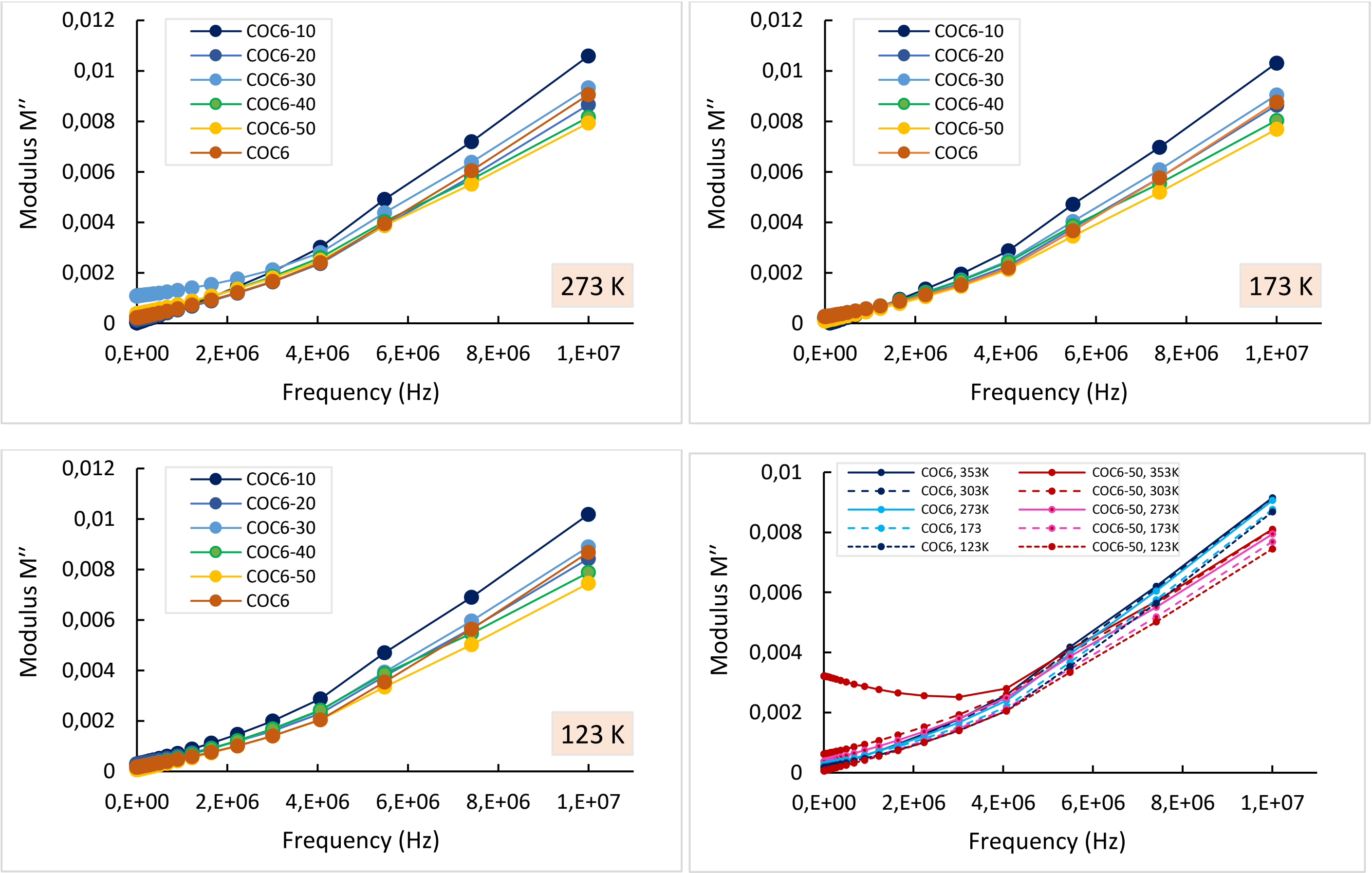


**Figure S4.** Trends of M’, in terms of frequency and temperatures

These observations are crucial for understanding the viscoelastic properties of both materials, especially in applications where temperature and frequency play critical roles, such as in cryogenic environments or dynamic loading conditions.
